# Favorable intermediate risk prostate cancer with biopsy Gleason score of 6

**DOI:** 10.1186/s12894-021-00827-2

**Published:** 2021-04-05

**Authors:** Jong Jin Oh, Hyungwoo Ahn, Sung Il Hwang, Hak Jong Lee, Gheeyoung Choe, Sangchul Lee, Hakmin Lee, Seok-Soo Byun, Sung Kyu Hong

**Affiliations:** 1 Department of Urology, Seoul National University Bundang Hospital, Seongnam, Korea; 2 Department of Urology, Seoul National University College of Medicine, Seoul, Korea; 3 Department of Radiology, Seoul National University Bundang Hospital, Seongnam, Korea; 4 Department of Radiology, Seoul National University College of Medicine, Seoul, Korea; 5 Department of Pathology, Seoul National University Bundang Hospital, Seongnam, Korea

**Keywords:** Prostate cancer, Intermediate risk group, MRI

## Abstract

**Background:**

To identify potential prognostic factors among patients with favorable intermediate risk prostate cancer with a biopsy Gleason score 6.

**Methods:**

From 2003 to 2019, favorable intermediate risk patients who underwent radical prostatectomy were included in this study. All patients were evaluated preoperatively with MRI. Using PI-RADS scores, patients were divided into two groups, and clinic-pathological outcomes were compared. The impact of preoperative factors on significant pathologic Gleason score upgrading (≥ 4 + 3) and biochemical recurrence were assessed via multivariate analysis. Subgroup analysis was performed in patients with PI-RADS ≤ 2.

**Results:**

Among the 239 patients, 116 (48.5%) were MRI-negative (PI-RADS ≤ 3) and 123 (51.5%) were MRI-positive (PI-RADS > 3). Six patients in the MRI-negative group (5.2%) were characterized as requiring significant pathologic Gleason score upgrading compared with 34 patients (27.6%) in the MRI-positive group (p < 0.001). PI-RADS score was shown to be a significant predictor of significant pathologic Gleason score upgrading (OR = 6.246, p < 0.001) and biochemical recurrence (HR = 2.595, p = 0.043). 10-years biochemical recurrence-free survival was estimated to be 84.4% and 72.6% in the MRI-negative and MRI-positive groups (p = 0.035). In the 79 patients with PI-RADS ≤ 2, tumor length in biopsy cores was identified as a significant predictor of pathologic Gleason score (OR = 11.336, p = 0.014).

**Conclusions:**

Among the patients with favorable intermediate risk prostate cancer with a biopsy Gleason score 6, preoperative MRI was capable of predicting significant pathologic Gleason score upgrading and biochemical recurrence. Especially, the patients with PI-RADS ≤ 2 and low biopsy tumor length could be a potential candidate to active surveillance.

## Background

Clinically localized prostate cancer can be managed with active surveillance (AS) [1]. Importantly, AS has emerged as a preferred initial management strategy for patients with low-risk (LR) PCa as it helps decrease the overtreatment of clinically indolent disease [2]. The safety and utility of AS in patients with LR PCa was confirmed and its use has rapidly increased worldwide. However, the utility of AS in patients with intermediate risk (IR) PCa remains unclear [3].

In the United States SEER (Surveillance, Epidemiology and End Results)-Medicare program, expectant management of LR PCa cases increased from 22 to 43% between 2004 and 2011; 15% to 18% of IR PCa cases were managed conservatively [4]. According to the National Comprehensive Cancer Network (NCCN) stratification, those classified within the favorable intermediate risk (FIR) group had better prognoses compared with those within the unfavorable intermediate risk (UFIR) group [5]; other studies revealed similar oncological results compared to LR PCa [6, 7]. Data from one long-term study revealed that a similar percentage of those in the IR group with a biopsy Gleason score of 6 and PSA between 10–20 ng/ml experienced 15-year metastatic-free survival compared to those with LR PCa (94% for both groups) [3]. Therefore, here we evaluated the potential utility of AS in individuals within the IR group, specifically those characterized as FIR.

Biochemical recurrence rates (BCR) following definitive primary treatment for IR PCa vary dramatically, with 5-year rates ranging from 2 to 70% [8–11]. Of note, current criteria for AS have a misclassification rate of between 15–30% [12–15]. Another study revealed that individuals with a bGS of 6 and a PSA between 10 and 20 ng/ml are at higher risk of pathologic Gleason score upgrading (PGU) and upstaging, making them poor candidates for AS [2]. A rate of 50% PGU has been reported in a variety of studies [16–18], suggesting that not all patients with bGS of 6 may be characterized appropriately.

Therefore, in this study, we characterized the outcomes of patients with FIR PCa who have a bGS of 6 and PSA between 10 and 20 ng/ml. Prognosis using MRI according to stratification was analyzed, and potential prognosticators were investigated among patients (and subgroups) with FIR PCa and clear MRI results.

## Methods

All data analysis was carried out in accordance with applicable laws and regulations described in the Declaration of Helsinki and approved by institutional review board approval (Seoul National University Bundang Hospital (B-2004–608-104), we reviewed the records of patients who underwent radical prostatectomy (RP) in a single tertiary hospital between November 2003 and April 2019. Among them, patients with FIR PCa, preoperative bGS of 6, PSA between 10 and 20 ng/ml and a percent of positive biopsy cores < 50% were finally enrolled. All patients received ≥ 12 core transrectal prostate biopsy (MR fusion) and preoperative prostate MRI.

Prostate MRI exams were conducted 2–6 weeks after transrectal ultrasound–guided biopsy and before surgery. Prostate MRI up to 2006 was acquired in a biparametric manner including T2-weighted image (T2WI) and diffusion-weighted image (DWI). Since 2007, multiparametric prostate MRI, including dynamic contrast-enhanced (DCE) image, have been obtained. As PI-RADS was released and revised during this period, and to minimize the bias that may occur due to accumulated MRI reading experience over time, an experienced uroradiologist blinded from relevant information reviewed the preoperative MRI of the included patients for this retrospective study. Final scores for each patient were assigned according to PI-RADS (version 2) standards on a 5-point scale [19, 20]**.** The probability of clinically significant cancer was defined as follows: 1 (very low), 2 (low), 3 (intermediate), 4 (high), and 5 (very high).

Enrolled patients were stratified into two group according to preoperative MRI findings—the MRI-negative group (PI-RADS < 4) and the MRI-positive group (PI-RADS ≥ 4). Baseline characteristics [ie, age, body mass index (BMI), prostate volume, PSA, percent of positive biopsy core (%), total tumor length in cores, percentage of total tumor of all cores] between the two groups were compared using the student-t and chi-square tests.

RP specimens were assessed as previously reported [21]. Pathological parameters [ie, pGS, PGU, extracapsular extension (ECE), seminal vesicle invasion (SVI), positive surgical margin (PSM), pathologic tumor volume] were compared. We defined significant PGU (SPGU) as pathologic Gleason score ≥ 4 + 3 from bGS 6. All biopsy and RP pathology was newly reviewed by one uro-pathologist. A multivariate analysis was performed to predict SPGU using preoperative parameters and the PI-RADS grouping. Median follow-up duration was 58 months. BCR was defined as postoperative PSA ≥ 0.2 ng/mL taken twice at least 6 weeks apart [22]. Multivariate Cox proportional hazard model was also performed to predict BCR using preoperative parameters. A subgroup analysis was conducted in patients whose preoperative MRI showed no significant lesions and identified other preoperative parameters that could be predictors of PGU.

## Results

In all, 239 patients with bGS of 6 and PSA between 10 and 20 ng/ml were enrolled in this study. Among them, 116 (48.5%) were placed into the MRI-negative group and 123 (51.5%) into the MRI-positive group. As shown in Table 1, those in the MRI-positive group had: i) a higher percentage of positive cores (28.51 vs. 20.42%, p = 0.003), ii) longer tumor lengths (0.51 vs. 0.32, mm, p < 0.001) and iii) percentage of total tumors of cores (32.02 vs. 20.76, %, p < 0.001). In the MRI-positive group, 64 (52.0%) and 59 (48.0%) of patients had PI-RADS scores of 4 and 5, respectively.

**Table 1:** Baseline characteristics who intermediate risk prostate cancer patients with biopsy Gleason score 6 and comparisons according to multi-parametric MRI finding

	Total	MR-negative	MR-positive	*p* value
Number	239	116 (48.5)	123 (51.5)	
Mean Age (years, ± SD)	65.79 ± 6.60	65.37 ± 7.15	66.19 ± 6.03	0.340
Mean BMI (± SD)	24.49 ± 2.64	24.41 ± 2.75	24.56 ± 2.55	0.678
Prostate volume	42.22 ± 18.43	45.27 ± 19.76	39.35 ± 16.65	0.013
PSA	13.51 ± 2.82	13.46 ± 2.86	13.56 ± 2.79	0.801
Median PSA	12.64	12.32	13.00	
Mean PSA density	0.37 ± 0.16	0.36 ± 0.17	0.39 ± 0.15	0.085
DM (%)	25 (10.5)	11 (9.5)	14 (11.4)	0.395
HTN (%)	113 (47.3)	54 (46.6)	59 (48.0)	0.464
Number of biopsy (%)				0.262
12	164 (68.6)	80 (69.0)	84 (68.3)	
≥ 13	75 (31.4)	36 (31.0)	39 (31.7)	
Mean percentage of positive core (%, ± SD)	24.44 ± 19.97	20.42 ± 17.09	28.51 ± 21.85	0.003
Mean tumor length (mm, ± SD)	0.41 ± 0.34	0.32 ± 0.26	0.51 ± 0.39	< 0.001
Mean percentage of total tumor of core (%, ± SD)	26.47 ± 22.50	20.76 ± 18.05	32.02 ± 24.96	< 0.001
PIRADS score (%)				
2	79 (33.1)	79		
3	37 (15.5)	37		
4	64 (26.8)		64	
5	59 (24.7)		59	

BMI; body mass index, PSA; prostate specific antigen, DM; diabetes mellitus, HTN; hypertension, PIRADS; Prostate Imaging Reporting and Data System,

Pathological outcomes are presented in Table 2. A total of 199 (83.3%) and 40 (16.7%) of patients, respectively demonstrated PGU and SPGU. The percentage of patients demonstrated PGU was significantly higher in the MRI-positive group (96.7%) compared with the MRI-negative group (69.0%) (p < 0.001); similar results were noted for SPGU (≥ 4 + 3) (27.6% vs 5.2% in the MRI-positive and MRI-negative group, respectively) (p < 0.001). Other parameters (ie, ECE, PSM, pathologic tumor volume) were also higher in the MRI-positive group compared with the MRI-negative group (all p < 0.001).

**Table 2:** Pathological outcomes after radical prostatectomy among favorable intermediate risk prostate cancer patients with biopsy Gleason score 6 and comparison according to multi-parametric MRI finding

	Total	MR-negative	MR-positive	p-value
Number	239	116 (48.5)	123 (51.5)	
Pathologic Gleason score (%)				< 0.001
6	40 (16.7)	36 (31.0)	4 (3.3)	
3 + 4	159 (66.5)	74 (63.8)	85 (69.1)	
4 + 3	38 (15.9)	5 (4.3)	33 (26.8)	
8	2 (0.8)	1 (0.9)	1 (0.8)	
Pathologic Gleason score upgrading (%)	199 (83.3)	80 (69.0)	119 (96.7)	< 0.001
Pathologic significant Gleason score upgrading (≥ 4 + 3) (%)	40 (16.7)	6 (5.2)	34 (27.6)	< 0.001
Extracapsular invasion (%)	50 (20.9)	10 (8.6)	40 (32.5)	< 0.001
Seminal vesicle invasion (%)	9 (3.8)	4 (3.4)	5 (4.1)	0.813
Bladder neck invasion (%)	6 (2.5)	1 (0.9)	5 (4.1)	0.116
Lymph node invasion	0			
Positive surgical margin (%)	53 (22.2)	13 (11.2)	40 (32.5)	< 0.001
Mean pathologic tumor volume (%, ± SD)	0.13 ± 0.14	0.07 ± 0.97	0.18 ± 0.16	< 0.001
Mean pathologic tumor volume (cc, ± SD)	5.16 ± 6.39	3.09 ± 3.96	7.11 ± 7.53	< 0.001

A multivariate analysis revealed that SPGU, PI-RADS (OR 6.246, 95% CI 2.400–16.255, p < 0.001) and percentage of total tumors of core (OR 1.049, 95% CI 1.014–1.086, p < 0.006) were significant predictors of SPGU (Table 3).

**Table 3:** Univariate and multivariate logistic regression analysis to predict pathologic significant Gleason score upgrading (≥ 4 + 3)

	Univariate analysis			Multivariate analysis		
	OR	95%CI	p-value	OR	95%CI	p-value
Age	1.000	0.950–1.053	0.998			
Bdoy mass index	1.092	0.958–1.245	0.186			
DM	0.937	0.303–2.892	0.909			
HTN	1.273	0.645–2.515	0.486			
Prostate volume	0.981	0.960–1.003	0.087			
PSA	0.936	0.824–1.063	0.310			
PSA density	2.755	0.352–21.536	0.334			
PIRADS (< 3 vs. ≥ 3)	6.940	2.788–17.275	< 0.001	6.246	2.400–16.255	< 0.001
Number of biopsy (12 vs. ≥ 13)	0.319	0.093–1.099	0.070			
Mean percentage of positive core	1.007	0.990–1.025	0.431			
Mean tumor length	3.667	1.493–9.005	0.005	0.133	0.014–1.285	0.081
Mean percentage of total tumor of core	1.027	1.012–1.041	< 0.001	1.049	1.014–1.086	0.006

**Fig. 1:**
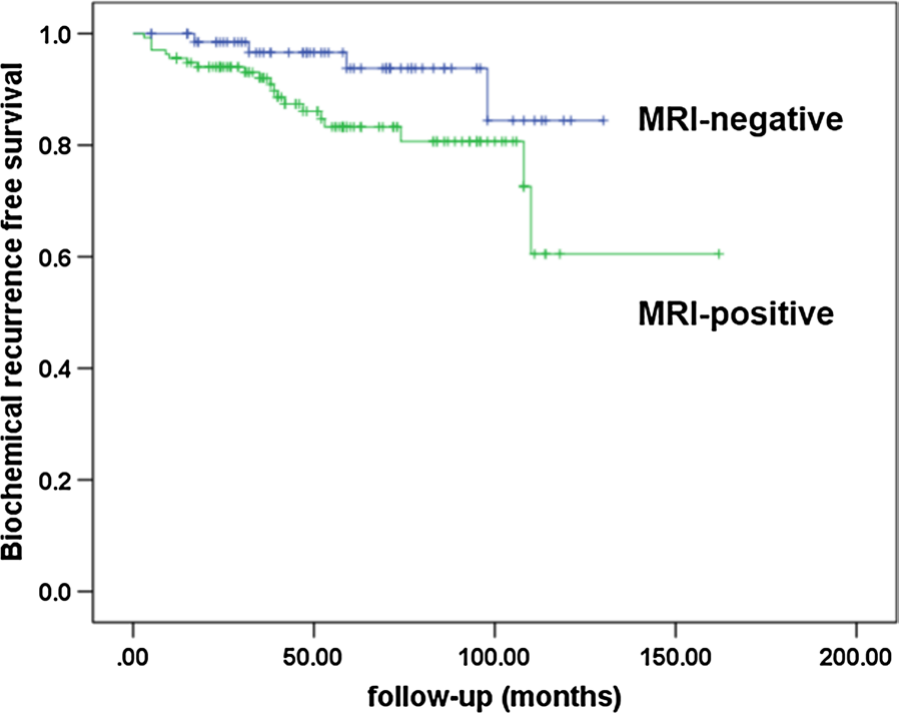
Kaplan-meier analysis of biochemical recurrence free survival according to MRI negative and positive

During a median follow-up of 58 months, 31 patients experienced BCR. 10-year BCR-free survival was achieved by 83.2% and 54.8% of those in the MRI-negative and MRI-positive groups, respectively (log rant test p = 0.027, Fig. 1). A mutivariate Cox proportional hazard model revealed that prostate volume (HR = 0.956, 95% CI 0.923–0.991, p = 0.013) and PI-RADS score (HR = 2.595, 95% CI 1.949–7.098, p = 0.043) were significant predictors of BCR (Table 4).

**Table 4:** Univariate and multivariate Cox proportional hazards analysis to predict biochemical recurrence after radical prostatectomy

	Univariate analysis			Multivariate analysis		
	HR	95%CI	p-value	HR	95%CI	p-value
Age	0.967	0.911–1.027	0.274			
Bdoy mass index	1.116	0.962–1.295	0.148			
DM	0.314	0.042–2.327	0.257			
HTN	1.854	0.793–4.335	0.154			
Prostate volume	0.953	0.921–0.986	0.005	0.956	0.923–0.991	0.013
PSA	1.043	0.907–1.199	0.555			
PSA density	14.810	2.307–95.077	0.004			
PIRADS (< 3 vs. ≥ 3)	3.002	1.024–8.800	0.035	2.595	1.949–7.098	0.043
Number of biopsy (12 vs. ≥ 13)	1.162	0.422–3.202	0.772			
Mean percentage of positive core	1.022	0.005–1.040	0.012	1.345	0.632–2.863	0.442
Mean tumor length	2.114	0.786–5.681	0.138			
Mean percentage of total tumor of core	1.012	0.997–1.027	0.117			

A subgroup analysis of the 79 patients with bGS of 6, PSA between 10-20 ng/ml and PI-RADS < 3, revealed that 48 (60.8%) and 10 (12.7%) required PGU and SPGU, respectively. Among this subgroup, BCR occurred in only 4 patients (5.1%). 10-year BCR-free survival was 84.4%. Multivariate analysis was used to predict PGU in this group and results are presented in Table 5. Tumor length as assessed from biopsy cores was a significant predictor of PGU (OR: 11.336, p = 0.014).

**Table 5:** Univariate and multivariate logistic regression analysis to predict pathologic Gleason score upgrading among PIRADS ≤ 2 patients

	Univariate analysis			Multivariate analysis		
	OR	95%CI	p-value	OR	95%CI	p-value
Age	1.021	0.958–1.088	0.523			
Bdoy mass index	1.100	0.925–1.307	0.280			
DM	0.804	0.198–3.261	0.760			
HTN	0.981	0.394–2.440	0.967			
Prostate volume	0.984	0.962–1.007	0.173			
PSA	0.991	0.844–1.164	0.915			
PSA density	1.811	0.688–4.764	0.329			
Number of biopsy (12 vs. ≥ 13)	0.615	0.196–1.933	0.406			
Mean percentage of positive core	3.400	1.010–11.451	0.048	0.962	0.905–1.022	0.206
Mean tumor length	6.500	2.114–19.987	0.001	11.336	1.630–78.845	0.014
Mean percentage of total tumor of core	3.274	1.210–8.861	0.020	1.042	0.976–1.112	0.220

## Discussion

In the current study, we investigated potential prognosticators of individuals with FIR PCa, a bGS of 6 and a PSA between 10 and 20 ng/ml. Among the 239 patients included: i) 10-year BCR-free survival was estimated to be 70.9%, ii) PGU and SPGU from bGS 6 were determined to be 83.3% and 16.7%, respectively, iii) and preoperative MRI findings were significantly predictors of PGU and SPGU. These results could help inform the selection of FIR patients who would be most appropriate for AS.

The possibility of using AS for patients in the IR risk group has been previously raised. Accumulating evidence suggests that FIR PCa may be similar biologically to LR PCa. For instance, a previous study revealed that there was no significant difference in BCR between those with FIR and LR PCa [5]. Additionally, this study reported 5-year progression-free survival (PFS) rates of 93% and 87% in LR and FIR risk groups, respectively (p = 0.054). These results are similar to what was observed in this study, namely a 10-year BCR-free survival rate of 70.9% in the FIR group.

Reports suggest that less aggressive IR cancer could be a potential candidate for AS. In particular, patients with Grade Group 1 (bGS 6) IR have been shown to have a low risk of progression to metastasis [23–26]. One large surveillance study (Sunnybrook in Toronto) reported data from a cohort of individuals receiving conservative treatment for IR PCa. Although the 15-year PCa metastatsis rate was 3.7 times higher in the IR risk group compared with the LR group, the presence of Gleason 7 cancer at initial diagnosis accounted for almost all of this increase in risk [3]. Patients with a bGS of 6 and PSA between 10 and 20 ng/ml had an estimated 15-year metastasis-free survival rate of 94%, a rate very similar to patients with LR PCa. This group highlighted that a PSA above 10 did not confer a significantly increased risk of metastasis in those without a cancer with a Gleason score of 4 [27]. Loeb et al. [28] revealed the patients with a bGS of 6 (Grade Group 1), PSA between 10 and 15 mg/ml and a PSA density less than 0.15 ng/ml did not significantly differ in adverse pathology findings when compared to those with LR PCa. As a result, the authors concluded that patients with bGS 6 IR PCa could be candidates for AS.

It should be noted, however, that other studies with contradictory findings have also been published. Aghazadeh et al. [5] conducted a large study (3,686 patients) which compared prognoses between FIR and LR PCa. The rate of adverse pathological findings in those with FIR was significantly higher when compared with those with lower risk PCa and significantly lower when compared with those with unfavorable intermediate risk PCa (27.4% vs 14.8% and 48.5%, respectively, each p < 0.001). In an Asian population study, the FIR group had significantly lower 5-year BCR-free survival when compared with the LR group (87.5 vs 93.5%; P = 0.002) [29]. These results could be caused by a discrepancy between bGS and pathological Gleason score. Yang et al. reported that 25.5% of patients with bGS 6 FIR PCa and PSA between 10 and 20 ng/ml required PGU and pathological upstaging [2]. Similarly, a report involving 359 men with bGS 6 and PSA between 10 and 20 ng/ml who underwent RP, revealed that 40.4% patients required PGU; among this group, 5% were upgraded into GS ≥ 8 [30]. Here, 83.3% of patients required PGU after RP, 16.7% had pathologic GS above 4 + 3. These results suggest that a proportion of patients with Gleason score 6 at preoperative biopsy may always require PGU.

Advances in software and hardware technology has led to the development of multi-parametric MRI for use in the detection of prostate cancer. Validation of this and other MRI-based tools have been summarized in guidelines published by the European Society of Urogenital Radiology (ESUR) along with a scoring system for PCa known as PI-RADS. Seo et al. revealed that PI-RADS can serve as a predictor of GS upgrading, with an estimated accuracy of 0.672–0.685 [1]. Another study of 126 cancer foci demonstrated that: i) PIRAD scores were 90% accurate at predicting Gleason score agreement between biopsy and pathologic GS (OR: 2.64, p < 0.001) and ii) MRI findings were capable of predicting PGU. Here, it was revealed that PI-RADS scores were a significant predictor of PGU (OR: 7.407, p < 0.001, data not shown) and SPGU (OR: 6.246, p < 0.001). Therefore, patients with FIR PCa, a bGS of 6 and a PI-RADS score ≤ 3 may be good candidates for AS. Among them, estimated 10-year BCR-free survival for patients with PCa and a PI-RADS score ≤ 2 was 84.4%, as good or better than the results reported from other studies of patients with LR PCa (66% to 88%) [31, 32]. Therefore, patients with FIR PCa with preoperative MRI (PI-RADS score ≤ 2) appear to be the ideal candidate for AS; furthermore, AS was seven safer in a subset of these patients with smaller biopsy tumor lengths.

This study does have several limitations that should be mentioned, primarily, the limited number of subjects and retrospective nature. Additionally, the PI-RADS score was assigned on biparametric MRI in patients included in earlier period. However, the diagnostic performance of the PI-RADS score of biparametric MRI is not reported to be inferior to that of multiparametric MRI [33]. Another limitation is the relatively high rate of PGU. The single pathologist who has a specialty for uro-oncology reviewed all of the specimens included in this study through International Society of Urological Pathology (ISUP) recommendation of modified Gleason score which announced in 2005 after handling by very thin sectioned. Regardless the extent of tumor, any Gleason pattern 4 was found in any section at radical prostatectomy specimen with 99% Gleason pattern 3, therefore Gleason score was 3 + 4. It was reason for high rate of PGU. In our results, 159 patients (79.9%) were pathologically upgraded to Gleason score 3 + 4 and only 38 patients (15.9%) to Gleason score 4 + 3 among 203 patients had experienced PGU after RP. The pathologic profiles of our participants appear to be relatively more aggressive (ie, higher PSA level, higher rate of high-grade disease) than those reported in western series. It is important to note that the rate of PSA screening in Asia is still not as high as in Western countries [34].

## Conclusions

Among the patients with FIR PCa, a bGS of 6 and a PSA of between 10-20 ng.ml, preoperative MRI was capable of predicting sPGU and BCR. Based on these results, we suggest that patients with FIR PCa who had a negative preoperative MRI and minimal tumor volume as assessed by biopsy are ideal candidates for AS.

## Data Availability

The full dataset generated and analyzed during the current study are available from the corresponding author on reasonable request.

## Abbreviations

PCa: Prostate cancer
bGS: Biopsy Gleason score
AS: Active surveillance
LR: Low risk
IR: Intermediate risk
SEER: Surveillance, Epidemiology and End Results
NCCN: National Comprehensive Cancer Network
FIR: Favorable intermediate risk
UFIR: Unfavorable intermediate risk
PSA: Prostate specific antigen
BCR: Biochemical recurrence
PGU: Pathologic Gleason score upgrading
MRI: Magnetic resonance imaging
RP: Radical prostatectomy
T2WI: T2-weighted image
DWI: Diffusion-weighed image
DCE: Dynamic contrast-enhanced
BMI: Body mass index
ECE: Extracapsular extension
SVI: Seminal vesicle invasion
PSM: Positive surgical margin
SPGU: Significant pathologic Gleason score upgrading
